# Large calcified renal artery aneurysm in the renal sinus misdiagnosed as an intrapelvic calculus followed by mistakenly performed PCNL: a case report

**DOI:** 10.1186/s12882-020-01998-0

**Published:** 2020-08-10

**Authors:** Chao Chen, Xuliang Wang, Fang Xin, Lugeng He, Kang Jiang, Jia Shao, Liping Xie

**Affiliations:** 1 Department of Urology, Affiliated Hangzhou First People’s Hospital, Zhejiang University School of Medicine, Hangzhou, China; 2 Department of Urology, The First Affiliated Hospital, Zhejiang University School of Medicine, Hangzhou, China

**Keywords:** Renal calculus, Renal artery aneurysm, Percutaneous nephrolithotomy, Embolization

## Abstract

**Background:**

Renal artery aneurysms (RAAs) are rare and usually asymptomatic, and some RAAs can be associated with calcifications, which may lead to misdiagnoses as renal calculi, which are then mistakenly treated.

**Case presentation:**

A 69-year-old female patient was admitted to the hospital with no discomfort and was diagnosed with a large right renal calculus. The ultrasound and computed tomography urography (CTU) scan suggested a large calculus in the right pelvis with hydrops of the kidney. Therefore, we chose percutaneous nephrolithotomy (PCNL) to treat the right renal calculus, but no calculi were found in the renal pelvis. When we removed the mucosa of the renal pelvis with a holmium laser, we observed a fluctuating unruptured aneurysm with calcification. Therefore, the previous diagnosis of a renal calculus was disregarded. The operation was stopped immediately, and then computed tomography (CT) angiography was performed, confirming the right renal aneurysm with calcification. Then, Renal artery aneurysm (RAA) coil embolization was performed. After a long-term follow-up, the patient recovered well.

**Conclusions:**

The RAA of this patient had calcific changes, which led us to errors in the diagnosis. Hence, it is very important for surgeons to effectively distinguish between renal calculi and aneurysms with ring-like calcifications. Our case report looks back at the thrilling situation during the operation and advises surgeons on how to deal with this situation properly.

## Background

Renal artery aneurysms (RAAs) are uncommon and usually asymptomatic, but some demonstrate clinical symptoms such as hypertension, abdominal pain, and haematuria and have a risk for rupture [1–3]. RAAs can be associated with calcifications, which may lead to misdiagnoses as renal calculi. We report a patient with a right calcified RAA misdiagnosed with a renal calculus who mistakenly underwent percutaneous nephrolithotomy (PCNL).

## Case presentation

A 69-year-old female patient who had a history of renal calculi and diabetes mellitus was admitted to the hospital because of a large right renal calculus found by urinary ultrasonographic examination.

**Fig. 1:**
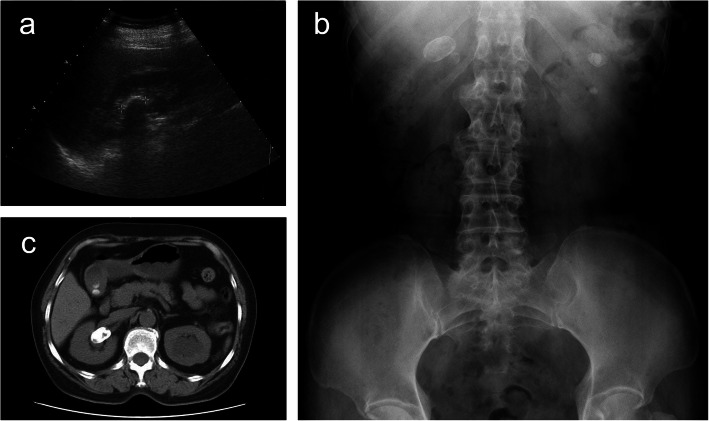
**a** Urinary ultrasonographic examination shows a hyperechoic focus in the right renal pelvis with an acoustic shadow. **b** KUB shows a large calculus in the right kidney and multiple calculi in the left kidney. **c** The non-contrast-enhanced CT image selected from the plain scan CTU sequence shows a calculus with a density of 508 HU and a size of 3 × 2.3 cm, with low-density lesions located in the right pelvis

The patient had no discomfort, and no positive signs were found on physical examination. The ultrasound showed that there was a large calculus in the right renal pelvis and multiple calculi in the left kidney, and both kidneys had hydronephrosis. The kidney-ureter-bladder radiograph (KUB) appearance supported the ultrasonographic findings. However, the core of the right renal calculus appeared to be radiolucent on plain films. The computed tomography urography (CTU) scan also suggested a calculus in the right pelvis with hydrops in the upper calyx of the kidney and left renal calculi with hydronephrosis (Fig. 1). However, when we analysed the CTU images for the second time, we found that there was a low-density lesion in the calculus on the non-contrast-enhanced CTU sequence, which we thought was a foreign body or infection, so empirical anti-infection therapy was provided.

**Fig. 2:**
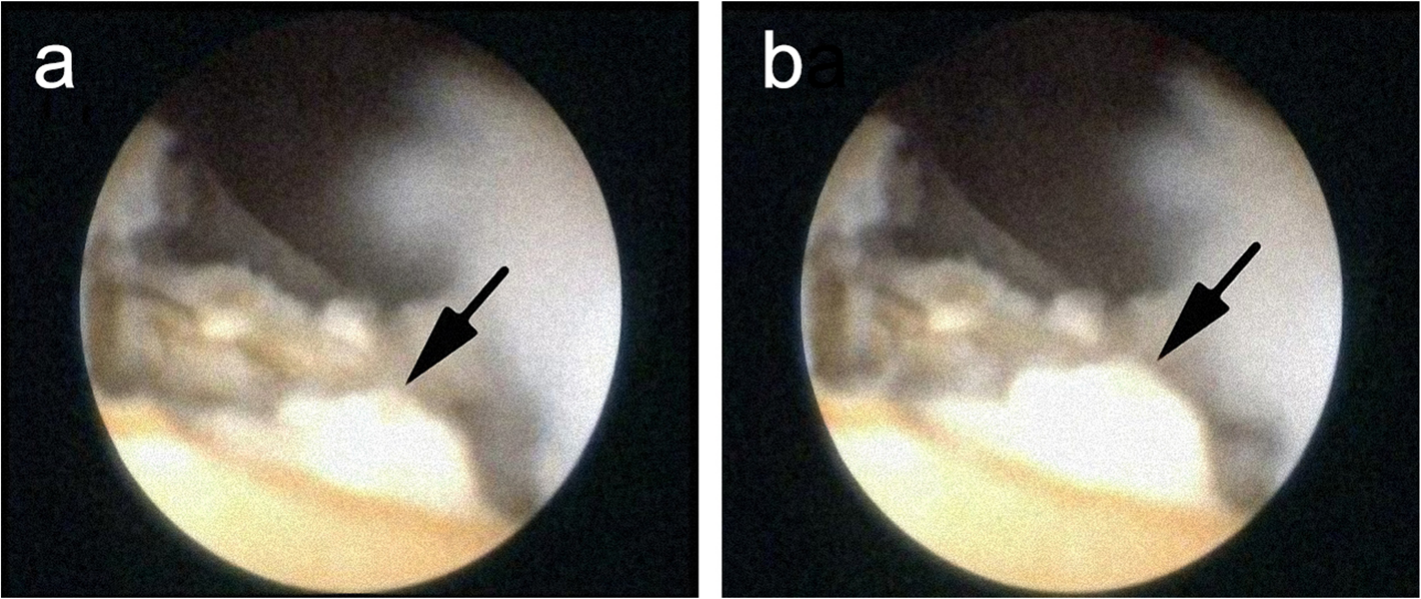
Images from a screenshot of the video show two different states of aneurysm pulsation (arrow). **a** The image shows the constricted state of aneurysm. **b** The image shows the expanded state of aneurysm

Then, PCNL was performed on the patient’s right renal pelvis calculus. We chose the posterior lower renal calyces for puncture and established the operation channel. No calculi were found in the renal pelvis or any calyces, but we could feel the calculus with the forceps under the mucosa of the renal pelvis, so we considered the calculus to be located in the diverticulum. Then, we removed the mucosa of the renal pelvis with a holmium laser until the calculus was observed. When cutting the surface of the calculus with a holmium laser, we observed a fluctuating unruptured aneurysm (Fig. 2). Therefore, the previous diagnosis of a renal calculus was disregarded, and a calcified renal artery aneurysm was considered.

**Fig. 3:**
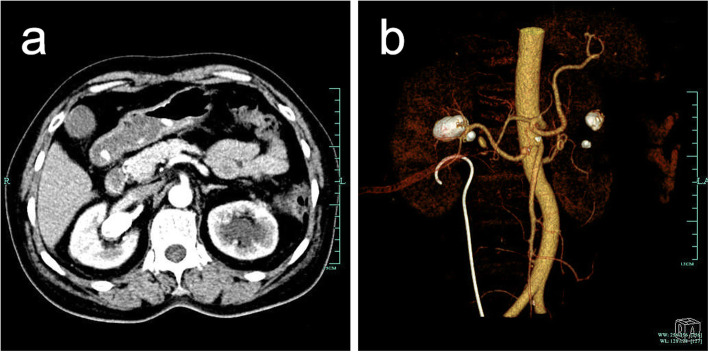
**a** The contrast-enhanced CT scan shows the right renal arterial branch penetrating into the aneurysm. **b** The coronal image and 3-dimensional reconstruction show multiple aneurysms in both kidneys. The large right RAA was located at the first bifurcation of the renal artery

**Fig. 4:**
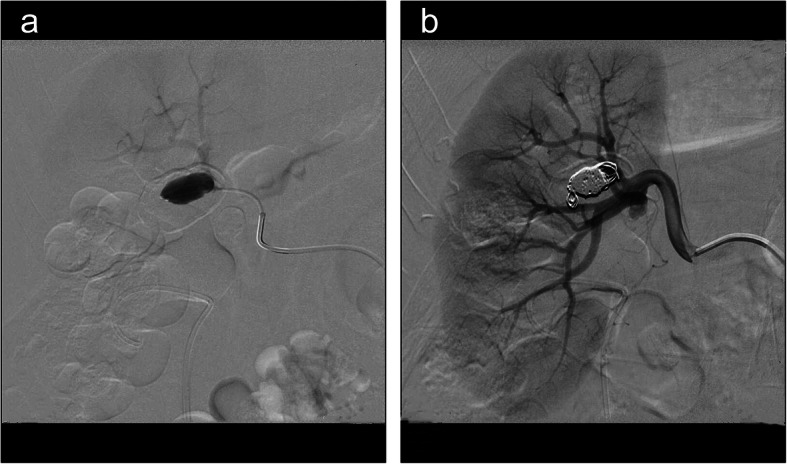
**a** Angiography image shows the right RAA. **b** Angiography image shows that the right RAA was treated with coil embolisation

The operation was stopped immediately, and the patient was transferred to the endovascular treatment department. The haemoglobin re-examination results were normal after PCNL; then, computed tomography (CT) angiography was performed, confirming the right renal aneurysm with calcification (Fig. 3). RAA coil embolization was performed under local anaesthesia on the 9th day after PCNL (Fig. 4).

After more than 1 year of follow-up, the patient recovered well.

## Discussion and conclusions

RAAs are defined as focal, isolated dilatations of all three layers of the arterial wall that measure > 1.5 times the diameter of the adjacent disease-free proximal arterial segment [4]. The estimated incidence of RAAs is very low, approximately 0.09% in the general population, but it may be as high as approximately 2.5% in the hypertensive population [5]. In recent years, with the increasing use of ultrasonography, CT, magnetic resonance imaging (MRI), and digital subtraction angiography (DSA) to image the renal artery, asymptomatic RAAs have been widely recognized and treated [6]. RAAs are usually asymptomatic, but symptomatic patients can have difficult-to-control hypertension, haematuria, and abdominal pain. A total of 56% of RAAs have calcific changes [7], so it is very important for us to effectively distinguish between renal calculi and aneurysms with ring-like calcifications.

Catheter-based arteriography is the gold standard for the diagnosis of RAAs, although CT is the most common contemporary non-invasive diagnostic modality, followed by MRI and ultrasonography. When an RAA is complicated by calcifications, it may be misdiagnosed as a renal calculus, especially if the calcified vessel wall of the renal arterial branch passes through the sinus [8]. In our case, both the ultrasound and CTU findings showed a calculus in the right pelvis. Although we found low-density lesions in the calculus on CT, we did not consider the rare occurrence of a calcified RAA. This is why PCNL was mistakenly performed. Fortunately, we followed the two principles of PCNL surgery and avoided directly rupturing the aneurysm. First, in the process of making the channel, we followed the direction of the target renal papilla for the puncture, rather than targeting the calculus. Second, in the process of lithotripsy, the surface of the stone was treated first, and the core was not always targeted. This case reminds us that if there are abnormal lesions in the renal calculus, the possibility of an aneurysm should be taken into account, and CT angiography, catheter angiography, or other examinations need to be completed for further diagnosis.

Currently, the accepted indications for RAA intervention include a size > 2 cm, female patients of childbearing age, ruptured RAAs, and symptoms, such as pain, haematuria, and medically refractory hypertension [9]. Surgical resection and endovascular repair are the main contemporary management strategies for RAAs. With the development of endovascular techniques and improvements in perioperative management, Cochennec F et al. [10] argues that endovascular treatment is a valid alternative to open repair, but patients are exposed to the risk of aneurysmal reperfusion. Therefore, the authors advise that endovascular repair should be considered as an elective first-line treatment for older patients, especially those with ruptured RAAs. We chose endovascular repair for our elderly patient in this case after a multidisciplinary team (MDT) meeting. The reasons were as follows. First, the size of the aneurysm was greater than 2 cm, although the patient had no symptoms. Second, the calcified shell of the aneurysm had been destroyed, and we considered that the wall of the aneurysm may be corroded by urine, which would increase the risk of aneurysm rupture. To date, no complications have occurred, including aneurysmal reperfusion.

The main reason that we performed the wrong treatment was that we made a misdiagnosis before the operation. We ignored the patient’s unusual CT and KUB imaging findings before PCNL. We report this case in order to avoid the recurrence of similar mistakes in the future and to learn something from this mistake. Our case suggests that for patients who are considered to have renal calculi clinically, we need to pay more attention to the patients’ CTU appearance, KUB presence, Doppler ultrasound imaging results, clinical symptoms and signs, histories of basic diseases such as hypertension, etc. We need to rule out the possibility of aneurysms associated with calcification if there are conditions such as an abnormal CT appearance, especially if low-density lesions are observed in the centre of the stone, if there are stones located in the path of the renal artery or its branches, if KUB shows ring-like calcifications in the region of the kidney, if Doppler ultrasound shows strong echo lesions around the renal sinus, if there is a lack of typical clinical symptoms and signs of urolithiasis, if there is a history of hypertension that is difficult to control, etc. We also need to pay attention to the patient’s sex and age group, as aneurysms are more common in older women [7]. Therefore, we strongly propose that CT angiography should be performed before considering surgical options for such renal calculi.

There are very few reported cases of RAAs misdiagnosed as renal calculi (Table. 1). To the best of our knowledge, unlike previously reported cases, this is the first reported case of a pulsating aneurysm that was observed during PCNL surgery. The case report looks back at the intense situation during surgery and advises surgeons on how to deal with this situation properly.

**Table 1:** Reported cases of RAAs misdiagnosed as renal calculi

Reference	Year	Age	Gender	Presentation	Misdiagnosis	Size (cm)	Hydro-nephrosis	Mis-treatment	Final treatment	Recurrence/Follow-up time
Chen SW et al. [8]	2013	51	Woman	Left lumbago	Left intrapelvic calculus	2.5	Yes	ESWL	Left nephrectomy	None/18 months
Sallami S et al. [11]	2014	42	Woman	Right-sided lumbar pain	Right intrapelvic calculus	1.1	No	ESWL	Vascular treatment	NA

*NA* not available, *ESWL* extracorporeal shock wave lithotripsy

## Data Availability

The datasets used and/or analysed during the current study are available from the corresponding author on reasonable request.

## Abbreviations

RAAs: Renal artery aneurysms
CTU: Computed tomography urography
PCNL: Percutaneous nephrolithotomy
CT: Computed tomography
KUB: Kidney-ureter-bladder radiograph
MRI: Magnetic resonance imaging
DSA: Digital subtraction angiography
MDT: Multidisciplinary team
